# Sex-specific associations of fat mass and muscle mass with cardiovascular disease risk factors in adults with type 2 diabetes living with overweight and obesity: secondary analysis of the Look AHEAD trial

**DOI:** 10.1186/s12933-022-01468-x

**Published:** 2022-03-15

**Authors:** Tasuku Terada, Jennifer L. Reed, Sol Vidal-Almela, Matheus Mistura, Kentaro Kamiya, Kimberley L. Way

**Affiliations:** 1 Exercise Physiology and Cardiovascular Health Lab, Division of Cardiac Prevention and Rehabilitation, University of Ottawa Heart Institute, 40 Ruskin Street, Ottawa, ON K1Y 4W7 Canada; 2 School of Epidemiology and Public Health, Faculty of Medicine, University of Ottawa, Ottawa, ON Canada; 3 School of Human Kinetics, Faculty of Health Sciences, University of Ottawa, Ottawa, ON Canada; 4 School of Allied Health Sciences, Kitasato University, Sagamihara, Japan; 5 Institute for Physical Activity and Nutrition, School of Exercise and Nutrition Sciences, Deakin University, Geelong, Australia

**Keywords:** Blood glucose, Exercise, Lifestyle intervention, Lipids, Obesity, Quality of life, Psychological health, Sarcopenia, Sex difference

## Abstract

**Background:**

Distinguishable sex differences exist in fat mass and muscle mass. High fat mass and low muscle mass are independently associated with cardiovascular disease (CVD) risk factors in people living with type 2 diabetes; however, it is unknown if the association between fat mass and CVD risk is modified by muscle mass, or vice versa. This study examined the sex-specific interplay between fat mass and muscle mass on CVD risk factors in adults with type 2 diabetes living with overweight and obesity.

**Methods:**

Dual-energy X-ray absorptiometry (DXA) measures were used to compute fat mass index (FMI) and appendicular muscle mass index (ASMI), and participants were separated into high-fat mass vs. low-fat mass and high-muscle mass vs. low-muscle mass. A two-way analysis of covariance (ANCOVA: high-FMI vs. low-FMI by high-ASMI vs. low-ASMI) was performed on CVD risk factors (i.e., hemoglobin A1C [A1C]; high-density lipoprotein cholesterol; low-density lipoprotein cholesterol; triglycerides; systolic and diastolic blood pressure; cardiorespiratory fitness, depression and health related-quality of life [HR-QoL]) at baseline and following a 1-year intensive lifestyle intervention (ILI) for females and males separately, with a primary focus on the fat mass by muscle mass interaction effects.

**Results:**

Data from 1,369 participants (62.7% females) who completed baseline DXA were analyzed. In females, there was a fat mass by muscle mass interaction effect on A1C (p = 0.016) at baseline. Post-hoc analysis showed that, in the low-FMI group, A1C was significantly higher in low-ASMI when compared to high-ASMI (60.3 ± 14.1 vs. 55.5 ± 13.5 mmol/mol, p = 0.023). In the high-FMI group, there was no difference between high-ASMI and low-ASMI (56.4 ± 12.5 vs. 56.5 ± 12.8 mmol/mol, p = 0.610). In males, only high-FMI was associated with higher A1C when compared to low-FMI (57.1 ± 14.4 vs. 54.2 ± 12.0 mmol/mol, p = 0.008) at baseline. Following ILI, there were significant fat mass by muscle mass interaction effects on changes in the mental component of HR-QoL in males.

**Conclusion:**

Considering that A1C predicts future CVD, strategies to lower A1C may be especially important in females with low fat and low muscle mass living with type 2 diabetes. Our results highlight the complicated and sex-specific contribution of fat mass and muscle mass to CVD risk factors.

**Supplementary Information:**

The online version contains supplementary material available at 10.1186/s12933-022-01468-x.

## Introduction

In the United States, 43.3% of middle-aged (40–59 years) females and 46.4% of middle-aged males were living with obesity between 2017 and 2018 [1], and up to 30% of community-dwelling females and males > 50 years of age were living with sarcopenia, an age-related decline in muscle mass [2]. The coexistence of obesity and sarcopenia is often termed sarcopenic obesity. Data from the National Health and Nutrition Examination Survey (NHANES) showed that 33.5% of females and 12.6% of males over 60 years of age are characterized as sarcopenic obese [3].

Type 2 diabetes is also prevalent among US adults, affecting 12.0% of females and 14.0% of males [4]. Excess body fat in type 2 diabetes, especially when distributed in the abdominal region, contributes to the clustering of cardiovascular disease (CVD) risk factors, including insulin resistance [5], glucose intolerance [6, 7], dyslipidemia [6], hypertension [7], low cardiorespiratory fitness (CRF) [8], depression and poor health-related quality of life (HR-QoL) [9]. While maintaining high muscle mass is critical to offset the negative effects of high fat mass on CVD risk factors [10], adults with type 2 diabetes show accelerated muscle mass loss [11]. Low muscle mass exacerbates insulin resistance [12], glycated hemoglobin A1C (A1C) [13], dyslipidemia [14], hypertension [14], CRF [15], depression [16] and HR-QoL [17], and predicts greater 10-year CVD incidences [18]. In type 2 diabetes, CVD accounts for ~ 65% of deaths [19]; the impact of diabetes on the risk of CVD mortality is greater for females than males [20]. While high fat mass and low muscle mass synergistically increase the risk of cumulative cardiovascular events in type 2 diabetes (e.g., stroke, myocardial infarction and death) [21], it remains unclear how fat mass and muscle mass simultaneously affect CVD risk factors.

Fat mass, fat distribution and muscle mass differ between sexes. Males generally have lower fat mass than females but have higher insulin resistance, blood glucose and lipid concentrations partly due to a greater amount of fat distributed in the abdominal region [10]. Females generally have lower absolute and body mass-adjusted muscle mass when compared to males [22], and females with type 2 diabetes are at heightened risk for muscle mass loss [11]. Lifestyle interventions including physical activity reduce fat mass while preserving muscle mass [23]. However, previous work has suggested that concurrent presentation of high fat mass and low muscle mass attenuates improvements in some CVD risk factors following exercise training, such as insulin resistance, fasting blood glucose and triglycerides in adults with type 2 diabetes [24]. It is unknown if the influence of fat mass and muscle mass on lifestyle-induced CVD risk improvements in type 2 diabetes is sex-specific.

Several studies have demonstrated that high fat mass and low muscle mass independently exacerbate CVD risks. However, the interplay between high fat mass and low muscle mass on CVD risk factors in adults with type 2 diabetes is less known. Given the sex-differences in body composition and risk of CVD mortality in type 2 diabetes, a sex-specific investigation of associations between body composition and CVD risk factors is warranted to understand how the unique body compositions of females and males contribute to CVD. The primary purpose of this study was to assess how fat mass and muscle mass would simultaneously predict CVD risk factors in females and males with type 2 diabetes. The secondary purpose was to examine how fat mass and muscle mass would simultaneously predict changes in CVD risk factors following a lifestyle intervention in females and males with type 2 diabetes. We hypothesized that, regardless of sex, high fat mass and low muscle mass would synergistically be associated with greater CVD risk factors and smaller improvements in CVD risk factors.

## Methods

### Study design

A secondary analysis was conducted on the longitudinal data collected in the Look AHEAD trial (ClinicalTrial.gov, number NCT00017953), the largest multicenter clinical trial designed to examine the effects of an intensive lifestyle intervention (ILI) on the prevention of CVD in individuals with type 2 diabetes [25]. The study protocol was approved by the Ottawa Health Science Network Research Ethics Board (Protocol #: 20200690-01H). The study was reported in accordance with the Strengthening the Reporting of Observational Studies in Epidemiology (STROBE) Statement [26].

From August 2001 to April 2004, adults with type 2 diabetes with a body mass index (BMI) ≥ 25 kg/m^2^ and between the ages of 45–76 years were recruited from 16 clinical sites across the United States. These participants were randomized (1:1) to either ILI or diabetes support and education (DSE). Details of ILI have been described elsewhere [25]. Briefly, ILI aimed at achieving and maintaining body mass loss of ≥ 7% by reducing caloric intake to 1200–1800 kcal/day and increasing caloric expenditure by ≥ 175 min/week of moderate intensity physical activity. ILI participants met with registered dieticians, behavioural psychologists, and exercise specialists weekly for the first 6 months (three group sessions and one individual session each month) and thrice monthly for the following 6 months (two group sessions and one individual session per month). At each session, the experts certified by Look AHEAD weighed participants, reviewed self-monitoring records completed by participants, and discussed behavioural strategies for weight loss, such as limiting times and places of eating; methods for exercising safely; and, reducing barriers to exercise [27]. To help participants achieve their dietary goals, meal replacement products, including Slim-Fast (Slim-Fast Foods Company, Englewood, NJ), Glucerna (Ross Laboratories, Columbus, OH), OPTIFAST (Novartis Nutrition, Fremont, MI), and HMR (Health Management Resources, Boston, MA) were provided at no cost. Participants were encouraged to increase caloric expenditure by modifying lifestyle behaviours, such as using stairs rather than elevators and walking rather than riding [28], and by accumulating bouts of physical activity at least 10 min in duration (e.g., brisk walking or similar aerobic activity). Participants were also provided with pedometers and encouraged to walk 10,000 steps each day.

Participants randomized to the DSE group received general information related to healthy eating and physical activity but did not receive the comprehensive components of the intervention nor specific strategies for weight loss. For this secondary analysis, data from phase I of the original trial [25] (baseline measures and measures taken one year following the intervention) were accessed because the lifestyle interventions were most strictly implemented and improvements in CVD risk factors were most prominent during this period.

### Participants

Of a total of 5,145 females and males with type 2 diabetes enrolled in the Look AHEAD trial [25], the subset of participants who completed dual-energy X-ray absorptiometry (DXA) at baseline were included in the analysis. All participants provided written informed consent, using a form approved by their local institutional board. This secondary data analysis was conducted on participants who consented to data sharing.

### Body composition phenotypes

Dual-energy X-ray absorptiometry was conducted at four sites using QDR-4500A fan beam densitometers (Hologic Inc., Bedford, MA, USA) at baseline and one-year following the interventions. DXA uses two-compartment models to distinguish fat mass and fat-free mass, and the fat-free mass can be subdivided into bone mineral and soft tissue. Skeletal muscle mass was calculated as the difference between fat-free mass and bone mineral content. Within-subject coefficient of variations for fat mass and muscle mass are 1.5% and 0.80%, respectively [29].

To quantify fat mass, fat mass index (FMI) was calculated by dividing whole body fat mass by height squared (m^2^) to account for different body sizes [30]. For muscle mass, appendicular skeletal muscle mass (ASM) was determined as the sum of muscle masses of both right and left extremities. To account for different body sizes, ASM index (ASMI) was calculated by adjusting the ASM for BMI as recommended by the Foundation for the National Institutes of Health (FNIH) [31]. Based on baseline FMI and ASMI, female and male participants were divided separately into high-fat mass (i.e. 50–100 FMI deciles) and low-fat mass (i.e. 0–49.99 FMI deciles) and into high-muscle mass (50–100 ASMI deciles) and low-muscle mass (0–49.99 ASMI deciles) [30] using sex-specific cutoffs.

### CVD risk factors

The outcome measures were CVD risk factors at baseline and following ILI, including A1C; fasting blood glucose; HDL-C; LDL-C; triglycerides; systolic and diastolic blood pressure (BP); CRF, depression and HR-QoL. Details of the blood glucose and lipid measures, the graded exercise test used for CRF assessment [32], the Beck Depression Inventory (BDI) used to assess depression severity (higher scores denote greater severity) [33], and the Medical Outcome Study Short Form-36 (SF-36) for physical component summary (PCS) and mental component summary (MCS) scores of HR-QoL (higher scores denote better QoL) [33] are described elsewhere.

### Statistical analysis

Data analyses were performed using IBM SPSS Statistics 27 for Windows (IBM Corp., Armonk, NY, USA). Data normality was tested with Kolmogorov–Smirnov test. BMI, A1C, fasting blood glucose, HDL-C, LDL-C, triglycerides, systolic BP, depression, PCS, MCS and CRF violated normality assumption. These data were normalized using a two-step approach [34]. Because the transformed data showed consistent results with non-transformed data, outputs using the non-transformed data are reported. Missing data were excluded from the analyses. Categorical variables are presented as frequencies and percentages, and continuous variables as mean ± standard deviation. Statistical significance was set at p < 0.05.

To assess the associations of fat mass and muscle mass with CVD risk factors at baseline, a two-way analysis of covariance (ANCOVA: high-FMI vs. low-FMI by high-ASMI vs. low-ASMI) was performed on baseline measures for females and males separately, with a primary focus on the fat mass by muscle mass interaction effects. The analyses were adjusted for age; race/ethnicity; income; duration of diabetes; and, prescribed medications to manage hyperglycemia, dyslipidemia, hypertension and depression. Details of medications are provided in Additional file 1: Tables S1 and S2. For females only, all analyses were additionally adjusted for the menopausal status. When significant interaction effects were found, we performed post-hoc analyses comparing high-ASMI and low-ASMI separately within high-FMI and low-FMI deciles. For sensitivity analysis, the truncal fat mass (calculated as the difference between total fat mass and appendicular fat mass) was used in place of FMI.

To examine if ILI was associated with improvements in CVD risk factors in the subset of participants included in the analysis, dependent t-tests were conducted for females and males separately to compare baseline and 1-year CVD risk factors. To assess the interaction between fat mass and muscle mass on changes in CVD risk factors, a two-way ANCOVA (high-FMI vs. low-FMI by high-ASMI vs. low-ASMI) on changes in blood glucose and lipids, BP, CRF, depression and HR-QoL was performed in females and males randomized to ILI. The analyses were adjusted for the same covariates as described above.

## Results

### Participants

Of 5,145 participants enrolled in the Look AHEAD trial, 1,369 (females, n = 858, 62.7%) completed DXA measures at baseline. A larger proportion of participants who completed DXA (i.e., those included in the analyses) were females when compared to those who did not undergo DXA assessment (62.5 vs. 57.1%, p < 0.001). Participants who completed DXA were younger (58 ± 6 vs. 59 ± 6 years old, p = 0.001); had significantly lower body mass (96.8 ± 16.7 vs. 102.7 ± 19.8 kg, p < 0.001), BMI (35.2 ± 5.3 vs. 36.2 ± 6.1 kg/m^2^, p < 0.001), waist circumference (111.0 ± 12.3 vs. 115.1 ± 14.4 cm, p < 0.001), triglyceride concentrations (175.2 ± 107.0 vs. 195.3 ± 133.9 mg/dL, p < 0.001) and MCS scores (53.1 ± 8.5 vs. 54.6 ± 7.8 points, p < 0.001); and, had higher depression scores (5.6 ± 5.1 vs. 5.3±4.8 point, p = 0.030) and CRF (7.5 ± 1.9 vs. 7.1 ± 2.0 metabolic equivalents [METs], p < 0.001) when compared to those who did not undergo DXA assessment.

### Baseline characteristics of females

Baseline characteristics of females (n = 858) are summarized in Table 1. When compared to females with low-FMI, those with high-FMI were significantly younger (56.8 ± 6.4 vs. 58.0 ± 6.0 years, p = 0.001) and had a shorter history of type 2 diabetes (5.9 ± 5.8 vs. 6.7 ± 6.1 years, p = 0.004). Females with low-ASMI were significantly older (57.7 ± 6.2 vs. 57.1 ± 6.6 years, p = 0.029) and had longer history of type 2 diabetes (6.8 ± 6.5 vs. 5.8 ± 5.4 years, p = 0.001) when compared to those with high-ASMI.

**Table 1:** Baseline characteristics of female participants

	High-FMI		Low-FMI		High-FMI vs Low-FMI, p-value	High-ASMI vs Low-ASMI, p-value	FMI by ASMI interaction, p-value
	High- ASMI (n = 157)	Low- ASMI (n = 272)	High- ASMI (n = 272)	Low- ASMI (n = 156)			
Age, year	57 (6)	59 (6)	57 (7)	59 (6)	**0.001**	**0.029**	0.809
Diabetes duration, year	6.0 (5.6)	7.4 (7.0)	6.4 (5.9)	7.1 (6.4)	**0.004**	**0.001**	0.631
ASMI, ASM/BMI	0.64 (0.05)	0.52 (0.04)	0.66 (0.07)	0.53 (0.04)	** < 0.001**	** < 0.001**	**0.022**
Fat mass index, kg/m^2^	17.5 (2.0)	18.9 (3.0)	12.1 (1.6)	13.0 (1.2)	** < 0.001**	** < 0.001**	0.131
Truncal fat mass, kg	25.0 (4.0)	25.4 (5.3)	17.4 (3.2)	16.8 (2.7)	** < 0.001**	0.059	0.098
Race/Ethnicity, n (%)					** < 0.001**	** < 0.001**	
White	104 (66.2)	132 (48.4)	144 (52.9)	46 (29.5)			
African American	37 (23.6)	31 (11.4)	46 (16.9)	4 (2.6)			
Hispanic	11 (7.0)	102 (37.4)	69 (25.4)	98 (62.8)			
Others	5 (3.2)	8 (2.9)	13 (4.8)	8 (5.1)			
Income, n (%)					0.414	** < 0.001**	
< $30,000	41 (26.1)	110 (40.3)	88 (32.4)	84 (53.8)			
$30,000—$59,999	52 (33.1)	83 (30.4)	79 (29.0)	39 (25.0)			
≥ $60,000	52 (33.1)	67 (24.5)	84 (30.9)	27 (17.3)			
Smoking, n (%)					0.407	0.786	
Never	92 (58.6)	172 (63.0)	179 (65.8)	103 (66.0)			
Past	57 (36.3)	90 (33.0)	82 (30.1)	46 (29.5)			
Current	7 (4.5)	11 (4.0)	11 (4.0)	7 (4.5)			
Medication, n (%)							
Anti-hyperglycemic	105 (69.1)	193 (69.7)	191 (68.7)	108 (71.5)	0.941	0.635	
Anti-dyslipidemic	64 (40.8)	89 (32.6)	99 (36.5)	58 (37.2)	0.352	0.508	
Anti-hypertensive	81 (53.3)	163 (58.8)	156 (56.1)	85 (56.3)	0.836	0.403	
Antidepressant	35 (22.3)	55 (20.1)	41 (15.1)	16 (10.3)	**0.013**	0.326	
Cardiovascular disease risk factors							
Body mass, kg	108.4 (10.5)	101.2 (14.2)	84.4 (9.8)	75.7 (7.5)	** < 0.001**	** < 0.001**	0.386
BMI, kg/m^2^	39.6 (3.5)	40.6 (4.5)	31.6 (2.8)	31.8 (2.4)	** < 0.001**	** < 0.001**	0.149
A1C, mmol/mol	56.4 (12.5)	56.5 (12.8)	55.5 (13.5)	60.3 (14.1)	0.427	0.214	**0.016**
A1C, %	7.3 (1.1)	7.3 (1.2)	7.2 (1.2)	7.7 (1.3)	0.427	0.214	**0.016**
Fasting blood glucose, mg/dL	153.3 (42.2)	156.5 (45.5)	151.9 (41.5)	162.8 (51.4)	0.549	0.225	0.238
HDL-C, mg/dL	47.2 (10.4)	45.9 (10.5)	47.4 (13.8)	47.2 (10.8)	0.077	0.388	0.503
LDL-C, mg/dL	116.3 (31.3)	117.5 (30.3)	115.8 (33.6)	121.2 (33.7)	0.137	0.062	0.165
Triglycerides, mg/dL	171.7 (98.5)	186.4 (93.9)	186.6 (107.5)	190.3 (98.6)	0.529	0.862	0.657
Systolic BP, mmHg	132.7 (18.7)	132.4 (17.3)	127.8 (16.9)	128.9 (16.7)	**0.006**	0.213	0.503
Diastolic BP, mmHg	69.3 (9.1)	67.3 (9.3)	68.7 (9.1)	66.4 (9.3)	0.402	0.341	0.765
CRF, MET	6.7 (1.5)	6.3 (1.3)	7.6 (1.8)	7.3 (1.6)	** < 0.001**	** < 0.001**	0.897
Depression, point	6.9 (5.8)	6.9 (5.4)	5.7 (4.9)	5.8 (4.7)	**0.004**	0.594	0.863
HR-QoL (PCS), point	46.4 (8.0)	45.8 (8.3)	49.6 (7.0)	49.2 (7.9)	** < 0.001**	0.081	0.965
HR-QoL (MCS), point	52.6 (8.5)	52.6 (8.7)	52.4 (9.0)	53.6 (8.1)	0.682	0.782	0.432

Data are reported mean (SD or n(%)). Values in boldface represent statistical significance (p < 0.05)

*ASMI* appendicular skeletal muscle mass index, *A1C* glycated hemoglobin A1C, *BMI* body mass index, *BP* blood pressure, *CRF* cardiorespiratory fitness, *FMI* fat mass index, *HR-QoL* health-related quality of life, *MCS* mental component summary, *MET* metabolic equivalent, and *PCS* physical component summary

**Fig. 1:**
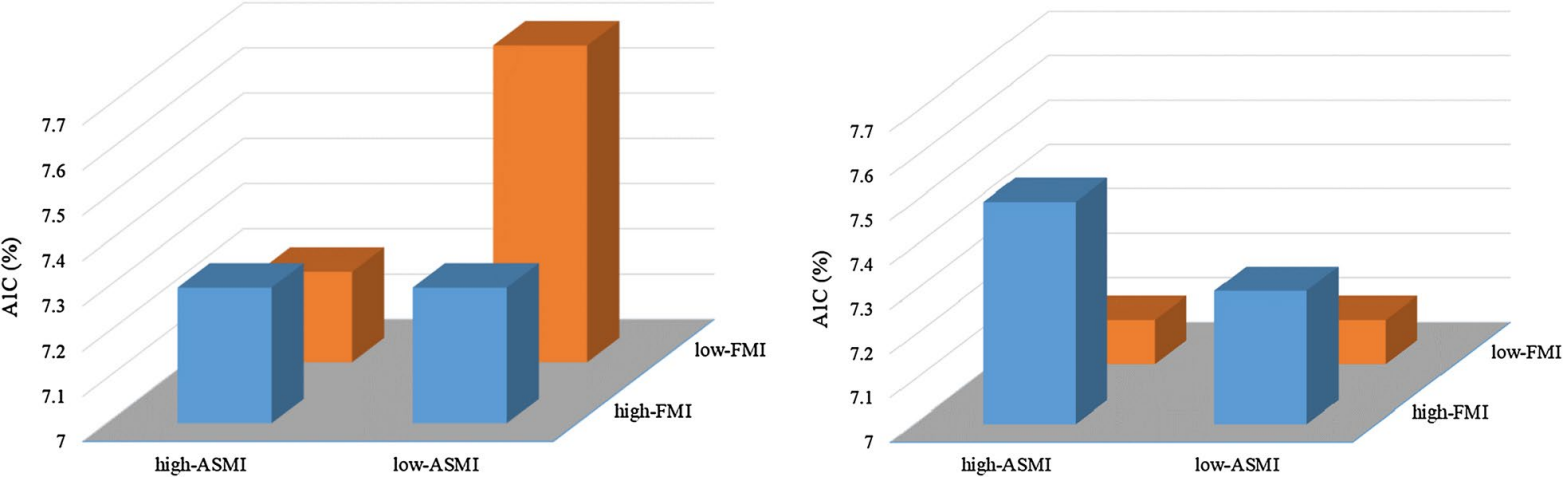
Glycated hemoglobin A1C concentration of females (left) and males (right) at baseline. In females, there was a significant FMI-by-ASMI interaction effect (p = 0.016). Post hoc analyses showed no significant difference between high-ASMI and low-ASMI in the high-FMI group (p = 0.610), whereas significantly higher A1C in low-ASMI when compared to high-ASMI in the low-FMI group (p = 0.023). In males, the high-FMI group had higher A1C when compared to low-FMI (p = 0.008)

The CVD risk factors at baseline are also summarized in Table 1. After adjusting for the covariates (i.e., age, duration of type 2 diabetes, race, income, prescribed medication and menopausal status), there was a significant FMI-by-ASMI interaction effect on A1C (interaction effect, p = 0.016, Fig. 1). Post-hoc analysis showed that, in the low-FMI group, A1C was significantly higher in low-ASMI when compared to high-ASMI (60.3 ± 14.1 vs. 55.5 ± 13.5 mmol/mol [7.7 ± 1.3 vs. 7.2 ± 1.2%], p = 0.023). In the high-FMI group, there was no difference between high-ASMI and low-ASMI (56.4 ± 12.5 vs. 56.5 ± 12.8 mmol/mol [7.3 ± 1.1 vs. 7.3 ± 1.2%] p = 0.610). Our sensitivity analysis using truncal fat mass instead of FMI showed consistent findings (interaction effect, p = 0.049). Females with high-FMI had higher systolic BP (132 ± 17 vs. 128 ± 16 mmHg, p = 0.006), lower CRF (6.4 ± 1.4 vs. 7.5 ± 1.7 METs, p < 0.001), higher depression (6.9 ± 5.5 vs. 5.7 ± 4.8 points, p = 0.004) and lower physical HR-QoL (i.e., PCS score: 46.0 ± 8.2 vs. 49.5 ± 7.4 points, p < 0.001) scores when compared to low-FMI. Females with low-ASMI had lower CRF when compared to high-AMSI (6.7 ± 1.5 vs. 7.3 ± 1.7 METs, p < 0.001).

### Baseline characteristics of males

Baseline characteristics and CVD risk factors of males (n = 511) are summarized in Table 2. There were no FMI-by-ASMI interaction effects on CVD risk factors at baseline. Males with high-FMI had higher A1C (57.1 ± 14.4 vs. 54.2 ± 12.0 mmol/mol [7.4 ± 1.3 vs. 7.1 ± 1.1%], p = 0.008, Fig. 1) and systolic BP (131 ± 16 vs. 127 ± 16 mmHg, p = 0.015), and lower CRF (7.5 ± 1.7 vs. 9.1 ± 2.2 METs, p < 0.001) and physical HR-QoL scores (PCS score: 47.4 ± 8.4 vs. 50.9 ± 6.6 points, p < 0.001) when compared to low-FMI. When comparing high- and low-ASMI, males with low-ASMI were significantly older (60.5 ± 6.4 vs. 59.7 ± 6.6 years old, p = 0.041) and had lower CRF (7.7 ± 1.8 vs. 9.0 ± 2.2 METs, p = 0.006).

**Table 2:** Baseline characteristics of male participants

	High-FMI		Low-FMI		High-FMI vs Low-FMI, p-value	High-ASMI vs Low-ASMI, p-value	FMI by ASMI interaction, p-value
	High-ASMI (n = 72)	Low-ASMI (n = 184)	High-ASMI (n = 184)	Low-ASMI (n = 71)			
Age, year	58.9 (6.5)	60.2 (6.5)	60.0 (6.6)	61.2 (6.2)	0.077	**0.041**	0.942
Diabetes duration, year	7.8 (5.6)	7.1 (6.9)	7.1 (6.5)	7.5 (6.7)	0.848	0.801	0.512
ASMI, kg/BMI	0.94 (0.06)	0.78 (0.06)	0.99 (0.08)	0.82 (0.04)	** < 0.001**	** < 0.001**	0.773
Fat mass index, kg/m^2^	11.6 (1.2)	13.5 (2.6)	8.3 (1.2)	9.2 (1.5)	** < 0.001**	** < 0.001**	**0.012**
Truncal fat mass, kg	2.2 (0.3)	2.4 (0.5)	1.5 (0.3)	1.6 (0.3)	** < 0.001**	**0.004**	0.112
Race/Ethnicity, n (%)					0.648	** < 0.001**	
White	51 (70.8)	125 (67.9)	130 (70.7)	57 (80.3)			
African American	3 (4.2)	14 (7.6)	8 (4.3)	4 (5.6)			
Hispanic	13 (18.1)	13 (18.5)	40 (21.7)	9 (12.7)			
Others	5 (6.9)	11 (6.0)	6 (3.3)	1 (1.4)			
Income, n (%)					0.553	** < 0.001**	
< $30,000	10 (13.9)	34 (18.5)	35 (19.0)	11 (15.5)			
$30,000—$59,999	21 (29.2)	49 (26.6)	38 (20.7)	20 (28.2)			
≥ $60,000	37 (51.4)	87 (47.3)	106 (57.6)	36 (50.7)			
Smoking, n (%)					0.189	0.908	
Never	27 (37.5)	73 (39.7)	73 (39.7)	31 (43.7)			
Past	37 (51.4)	100 (54.3)	104 (56.5)	38 (53.5)			
Current	8 (11.1)	11 (6.0)	7 (3.8)	2 (2.8)			
Medication, n (%)							
Anti-hyperglycemic	49 (69.0)	142 (77.6)	133 (72.3)	47 (64.4)	0.191	0.534	
Anti-dyslipidemic	41 (56.9)	87 (47.3)	96 (52.2)	30 (42.3)	0.565	0.452	
Anti-hypertensive	46 (64.8)	127 (69.4)	112 (60.9)	46 (63.0)	0.117	0.184	
Antidepressant	11 (15.3)	17 (9.2)	17 (9.2)	8 (11.3)	0.114	0.459	
Cardiovascular disease risk factors							
Weight, kg	116.6 (9.4)	110.9 (15.2)	111.0 (15.2)	90.3 (9.8)	** < 0.001**	** < 0.001**	0.501
BMI, kg/m^2^	35.8 (2.4)	37.5 (4.2)	30.8 (2.4)	31.4 (2.8)	** < 0.001**	** < 0.001**	0.051
A1C, mmol/mol	58.0 (13.0)	56.8 (14.9)	54.3 (11.8)	54.1 (12.6)	**0.008**	0.263	0.846
A1C, %	7.5 (1.2)	7.3 (1.4)	7.1 (1.1)	7.1 (1.2)	**0.008**	0.263	0.846
Fasting glucose, mg/dL	160.9 (44.1)	158.2 (50.6)	154.1 (43.0)	154.1 (49.7)	0.129	0.876	0.801
HDL-C, mg/dL	36.8 (8.1)	38.1 (8.6)	37.4 (8.9)	36.7 (9.7)	0.382	0.835	0.520
LDL-C, mg/dL	105.4 (29.9)	108.7 (31.5)	104.9 (27.1)	109.3 (34.7)	0.640	0.280	0.933
Triglycerides, mg/dL	203.6 (118.9)	207.3 (135.0)	185.6 (105.5)	207.9 (156.8)	0.946	0.152	0.448
Systolic BP, mmHg	131.0 (16.3)	131.1 (16.9)	129.0 (16.7)	123.6 (16.8)	**0.015**	0.211	0.060
Diastolic BP, mmHg	76.1 (7.8)	72.2 (9.2)	73.8 (8.6)	70.8 (8.2)	**0.039**	** < 0.001**	0.788
CRF, MET	7.9 (1.8)	7.3 (1.6)	9.4 (2.2)	8.6 (2.1)	** < 0.001**	**0.006**	0.867
Depression, point	4.8 (4.1)	5.5 (5.0)	4.0 (4.4)	5.7 (5.0)	0.373	0.107	0.715
HR-QoL (PCS), point	47.5 (9.0)	47.3 (8.2)	51.4 (6.5)	49.7 (6.8)	** < 0.001**	0.379	0.754
HR-QoL (MCS), point	52.6 (9.1)	53.6 (8.7)	54.2 (7.2)	53.9 (8.1)	0.299	0.565	0.727

Data are reported mean (SD or n(%)). Values in boldface represent statistical significance (p < 0.05)

*ASMI* appendicular skeletal muscle mass index, *A1C* glycated hemoglobin A1C, *BMI* body mass index, *BP* blood pressure, *CRF* cardiorespiratory fitness, *FMI* fat mass index, *HR-QoL* health-related quality of life, *MCS* mental component summary, *MET* metabolic equivalent, and *PCS* physical component summary

### Changes in CVD risk factors in females

Of 858 females, 410 were randomized to ILI. Dependent t-tests showed a significant reduction in FMI, increase in ASMI, and improvements in all CVD risk factors following ILI (all p < 0.05). Changes in CVD risk factors according to FMI and ASMI are summarized in Table 3. No FMI-by-ASMI interaction effect was observed on changes in CVD risk factors. Females with high-FMI at baseline showed greater reductions in body mass, BMI, and depression score (all p < 0.05). Between low- and high-ASMI, females with low-ASMI at baseline showed a greater decrease in systolic BP (-9 ± 17 vs. -7 ± 17 mmHg, p = 0.045) when compared to high-ASMI.

**Table 3:** Changes in cardiovascular disease risk factors of females following intensive lifestyle intervention

	High-FMI		Low-FMI		High-FMI vs Low-FMI, p-value	High-ASMI vs Low-ASMI, p-value	FMI by ASMI interaction, p-value
	High-ASMI (n = 157)	Low-ASMI (n = 272)	High-ASMI (n = 272)	Low-ASMI (n = 156)			
Body mass, kg	− 9.2 (6.7)	− 9.9 (6.0)	− 6.7 (5.8)	− 6.1 (4.7)	** < 0.001**	0.698	0.304
BMI, kg/m^2^	− 3.4 (2.5)	− 4.0 (2.4)	− 2.6 (1.1)	− 2.6 (2.0)	** < 0.001**	0.609	0.203
A1C, mmol/mol	− 27.4 (10.3)	− 30.5 (10.8)	− 29.5 (10.7)	− 32.5 (12.6)	0.275	0.097	0.937
A1C, %	− 0.36 (0.9)	− 0.65 (1.0)	− 0.55 (1.0)	− 0.82 (1.2)	0.275	0.097	0.937
Fasting blood glucose, mg/dL	− 14.9 (45.3)	− 31.0 (49.8)	− 24.5 (33.8)	− 26.7 (55.3)	0.622	0.669	0.165
HDL-C, mg/dL	3.6 (7.9)	2.5 (6.9)	4.4 (8.5)	4.0 (6.9)	0.472	0.137	0.761
LDL-C, mg/dL	− 5.9 (31.1)	− 3.3 (29.5)	− 1.7 (25.3)	− 2.3 (37.4)	0.896	0.594	0.467
Triglycerides, mg/dL	− 30.6 (143.9)	− 22.8 (85.2)	− 27.5 (76.2)	− 26.7 (97.0)	0.828	0.659	0.441
Systolic blood pressure, mmHg	− 9 (19)	− 11 (18)	− 5 (17)	− 7 (15)	0.183	**0.045**	0.907
Diastolic blood pressure, mmHg	− 3 (9)	− 4 (9)	− 2 (8)	− 2 (8)	0.448	0.251	0.321
Cardiorespiratory fitness, MET	1.6 (1.8)	1.4 (1.8)	1.8 (2.5)	1.4 (2.5)	0.755	0.264	0.596
Depression, point	− 3.1 (5.1)	− 2.6 (5.4)	− 1.4 (5.1)	− 1.4 (4.4)	**0.027**	0.576	0.638
HR-QoL (PCS), point	3.1 (7.0)	2.5 (9.7)	0.7 (6.8)	3.2 (7.5)	0.770	0.977	0.050
HR-QoL (MCS), point	0.6 (10.4)	1.2 (10.6)	1.4 (10.0)	1.2 (7.4)	0.793	0.526	0.772

Data are reported mean (SD). Values in boldface represent statistical significance (p < 0.05)

*ASMI* appendicular skeletal muscle mass index, *A1C* glycated hemoglobin A1C, *BMI* body mass index, *FMI* fat mass index, *HR-QoL* health-related quality of life, *MCS* mental component summary, *MET* metabolic equivalent, *PCS* physical component summary

### Changes in CVD risk factors in males

Of 511 males, 235 were randomized to ILI. Dependent t-tests showed a significant decrease in FMI and improvements in all CVD risk factors (all p < 0.05). However, ASMI decreased significantly over time (− 0.13 ± 0.22, p < 0.001). Changes in CVD risk factors according to FMI and ASMI are summarized in Table 4. Males with high-FMI at baseline experienced a significantly greater decrease in A1C (− 31.8 ± 13.8 vs. − 30.2 ± 9.6 mmol/mol [− 0.8 ± 1.3 vs. − 0.6 ± 0.9%], p = 0.038) and fasting blood glucose concentration (− 30.2 ± 55.6 vs. − 16.7 ± 41.0 mg/dL, p = 0.008) when compared to those with low-FMI at baseline. Between low- and high-ASMI, reductions in body mass and BMI were greater in males with low-ASMI than high-ASMI (both p < 0.05).

**Table 4:** Changes in cardiovascular disease risk factors of males following intensive lifestyle intervention

	High-FMI		Low-FMI		High-FMI vs Low-FMI, p-value	High-ASMI vs Low-ASMI, p-value	FMI by ASMI interaction, p-value
	High-ASMI (n = 72)	Low-ASMI (n = 184)	High-ASMI (n = 184)	Low-ASMI (n = 71)			
Body mass, kg	− 9.5 (8.1)	− 12.0 (8.3)	− 8.8 (5.7)	− 9.0 (7.1)	0.097	**0.041**	0.557
BMI, kg/m^2^	− 3.0 (2.7)	− 4.0 (2.7)	− 2.8 (1.8)	− 3.1 (2.4)	0.104	**0.012**	0.612
A1C, mmol/mol	− 34.4 (13.6)	− 31.1 (13.9)	− 31.1 (9.5)	− 27.9 (9.5)	**0.038**	0.095	0.824
A1C, %	− 1.0 (1.2)	− 0.7 (1.3)	− 0.7 (0.9)	− 0.4 (0.9)	**0.038**	0.095	0.824
Fasting blood glucose, mg/dL	− 40.2 (47.5)	− 27.4 (57.9)	− 17.2 (43.3)	− 15.3 (34.9)	**0.008**	0.393	0.334
HDL-C, mg/dL	5.4 (5.2)	3.7 (6.8)	4.7 (7.6)	4.6 (7.3)	0.966	0.333	0.309
LDL-C, mg/dL	− 6.7 (25.0)	− 5.7 (24.7)	− 6.0 (25.7)	− 3.4 (31.7)	0.668	0.391	0.758
Triglycerides, mg/dL	− 59.4 (133.3)	− 65.0 (142.1)	− 33.0 (167.1)	− 47.4 (87.0)	0.240	0.553	0.758
Systolic blood pressure, mmHg	− 5 (18.0)	− 10 (18)	− 8 (16)	− 5 (14)	0.845	0.591	0.212
Diastolic blood pressure, mmHg	− 4 (7)	− 3 (8)	− 4 (9)	− 3 (7)	0.934	0.167	0.727
Cardiorespiratory fitness, MET	2.4 (2.1)	1.9 (2.2)	1.5 (2.8)	2.3 (2.7)	0.607	0.436	0.058
Depression, point	− 1.8 (4.7)	− 2.0 (4.5)	− 0.6 (4.9)	− 2.1 (4.4)	0.150	0.670	0.358
HR-QoL (PCS), point	− 0.2 (12.4)	3.8 (7.2)	1.9 (6.1)	2.9 (7.6)	0.862	0.058	0.112
HR-QoL (MCS), point	5.2 (9.4)	1.2 (9.5)	0.1 (6.6)	3.5 (6.5)	0.113	0.236	**0.003**

Data are reported mean (SD). Values in boldface represent statistical significance (p < 0.05)

*ASMI* appendicular skeletal muscle mass index, *A1C* glycated hemoglobin A1C, *BMI* body mass index, *HR-QoL* health-related quality of life, *MCS* mental component summary, *MET* metabolic equivalent, *PCS* physical component summary

**Fig. 2:**
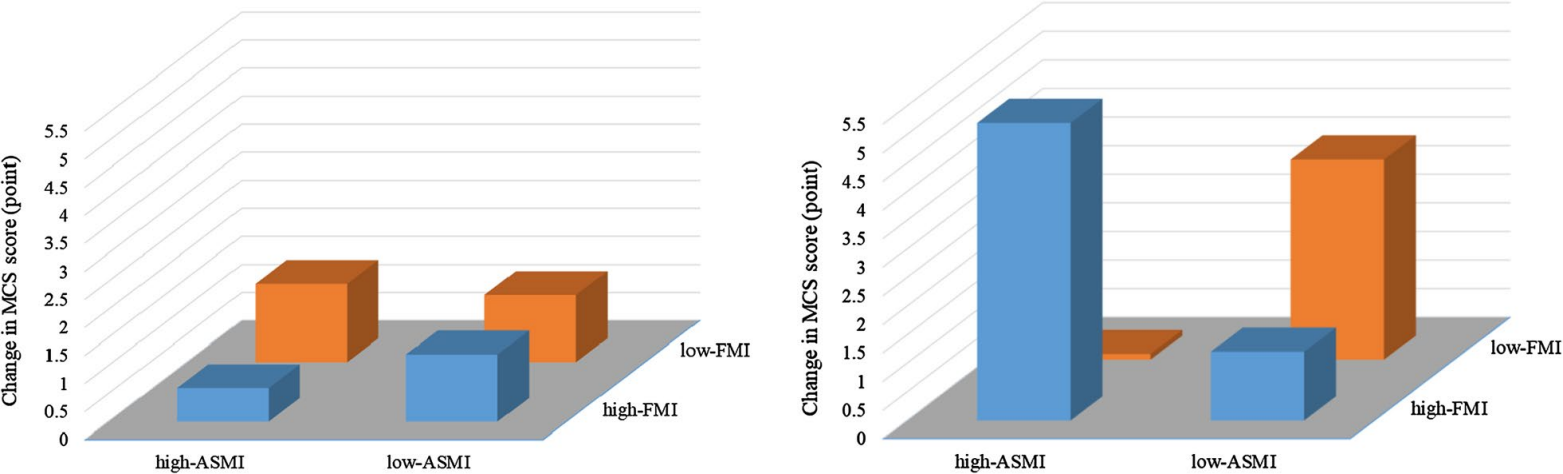
Changes in mental component summary (MCS) scores following intensive lifestyle intervention in females (left) and males (right). In females, there were no differences in changes in MCS scores associated with different body composition phenotypes. In males, there was an FMI-by-ASMI interaction effect on changes in MCS scores (p = 0.003). Post hoc analyses showed a greater increase in MCS scores in high-ASMI when compared to low-ASMI in the high-FMI group. In the low-FMI group, MCS scores increased more in low-ASMI when compared to high-ASMI

There was a significant FMI-by-ASMI interaction effect on the changes in mental HR-QoL (i.e., MCS scores: interaction effect, p = 0.003, Fig. 2). Post hoc analyses showed a significantly greater increase in MCS scores in males with high-ASMI when compared to low-ASMI in high-FMI at baseline (5.2 ± 9.4 vs. 1.2 ± 9.5 points, p = 0.016). In contrast, the MCS score increased more in males with low-ASMI when compared to high-ASMI in low-FMI at baseline (3.5 ± 6.5 vs. 0.1 ± 6.6 points, p = 0.042). Our sensitivity analysis using the truncal fat mass did not confirm the FMI-by-ASMI interaction effect (p = 0.197).

## Discussion

The combination of high fat mass and low muscle mass increase the risk of CVD incidences [21]. This study assessed the sex-specific interplay between fat mass and muscle mass on CVD risk factors in adults with type 2 diabetes living with overweight or obesity. Contrary to our hypothesis that high fat mass and low muscle mass would synergistically worsen CVD risks, we found that, in females, low fat mass and low muscle mass were synergistically associated with higher A1C. This result suggests that low muscle mass has deleterious effects on A1C only when combined with low fat mass in females. In males, no significant influence of muscle mass on A1C was found and high-fat mass was significantly associated with higher A1C. Regarding the changes in CVD risk factors following ILI, we found a significant fat mass by muscle mass interaction effect on ILI-induced changes in MCS scores in males.

No previous study has examined the sex-specific interplay between fat mass and muscle on A1C. This is the first study to report synergistic exacerbation of A1C by low fat mass and low muscle mass among females with type 2 diabetes living with overweight or obesity. This finding, although unexpected, was similar to previous large scale retrospective studies demonstrating significantly higher A1C in underweight (BMI < 18.5 kg/m^2^, 9.6 ± 2.7%) when compared to higher BMI (≥ 27.5 kg/m^2^, 8.2 ± 1.8%, p < 0.001) [35], and non significantly higher A1C in females with lower BMI (20 to 25 kg/m^2^, 8.1 ± 2.2%) when compared to higher BMI categories (e.g., ≥ 30 kg/m^2^, ≤ 7.9%) [36]. Because BMI reflects the sum of both fat mass and muscle mass, lower BMI in these studies likely reflects low fat mass and low muscle mass. Every 0.5% increment in A1C is positively associated with increased risk of CVD (RR:1.29, 95% CI 1.11 to 1.50) [37], and those with A1C ≥ 7.5% face elevated future risks of total CVD (HR: 1.82, 95% CI: 1.01–3.26) and all cause mortality (HR: 2.45, 95% CI: 1.45–4.14) when compared to A1C < 5.5% [38]. Consequently, the mean A1C of 7.7% observed in females with low fat mass and low muscle compared to A1C of ≤ 7.3% in the other body composition phenotypes may be clinically meaningful. Interventions that target A1C and low muscle mass may be necessary for females with low fat mass and low muscle mass to avoid future complications.

The mechanism behind synergistic exacerbation of A1C by low fat mass and low muscle mass in females is unclear. It is possible that higher systemic inflammation contributed to muscle mass loss and higher A1C [39]. Insulin resistance may have also increased A1C and decreased stimulation of protein synthesis while promoting activation of protein degradation [40]. Insulin resistance is generally higher in those with more fat distributed in the android region and visceral adipose tissue is more strongly linked to insulin resistance in females [41]. We conducted sensitivity analysis using truncal fat mass and confirmed that lower truncal fat mass and lower muscle mass are synergistically associated with exacerbated A1C in females. However, our data did not allow us to distinguish visceral and subcutaneous fat amounts. Interestingly, while fat mass was positively associated with A1C in males, in females there were no significant associations between baseline A1C and FMI, body mass or BMI (all p > 0.05, results not shown). Further studies simultaneously examining sex-specific muscle mass, fat mass and fat distribution are warranted to elucidate the intricate interplay between fat mass and muscle mass and to develop more tailored programs to manage unique body compositions of both sexes.

With regards to the other CVD risk factors at baseline, we found that high fat mass was associated with worse systolic BP, CRF, depression and PCS scores regardless of sex. We also found that low muscle mass was associated with lower CRF in both sexes. These findings are in line with a previous study [15] and highlight the negative effects of high fat mass on CVD risks factors. Because lower CRF is associated with the development of CVD [42] and predicts mortality in type 2 diabetes [43], our results suggest that high fat mass and low muscle mass in adults with type 2 diabetes are independently associated with higher risk of future CVD.

Following ILI, consistent to the previous report including all participants (N = 5,145) [25], we found significant improvement in body compositions in females (i.e., decreased fat mass and increased muscle mass). Such improvements were accompanied by overall improvements in CVD risk factors. Between high-FMI and low-FMI at baseline, depression improved significantly more in females with high-FMI at baseline when compared to females with low-FMI. A greater decrease in fat mass in females with high-FMI at baseline when compared to low-FMI (− 5.0 ± 5.1 vs. 1.1 ± 4.3 kg/m^2^, p < 0.001) may have positively affected societal stigmatization and self-esteem [44] and resulted in enhanced reduction in depression scores [45]. The systolic BP decreased significantly more in females with low-ASMI when compared to high-ASMI (− 10 ± 17 vs. − 7 ± 18 mmHg, p = 0.045). However, this difference may be of little clinical importance. Overall, our results highlight positive effects of ILI on CVD risk factors in females regardless of fat mass or muscle mass before participating in ILI.

In males, weight loss was accompanied by significant decreases in FMI and ASMI. The reduction in FMI was significantly greater in males with high-FMI at baseline when compared to males with low-FMI, which may explain greater decreases in A1C and fasting blood glucose in high-FMI compared to low-FMI. The loss of muscle mass at 1 year corresponds to previous findings [46]. Loss of muscle mass only in males is in line with a previous study reporting that males lose greater muscle mass than females when total body mass is reduced [47]. ILI represented by exercise and hypocaloric diet is a more effective intervention for weight loss than either of the interventions alone; however, the skeletal muscle mass is not completely preserved [23]. Our results suggest that males require strategies to maintain muscle mass when ILI targeting weight loss is implemented. Considering blunted protein synthetic response to the anabolic stimuli in individuals with obesity, they may have higher protein needs compared to leaner individuals [23]. Addition of resistance exercise and increased protein intake to counteract the loss of muscle mass may be more important in males aiming to lose weight through lifestyle interventions.

In males, there was a significant fat mass by muscle mass interaction effect on the mental component of HR-QoL (i.e., MCS scores). There is no clear explanation for such different degrees of improvements. However, when we examined subscales of HR-QoL, mental health showed the same interaction effect (p = 0.001), and social function also showed the same patterns of improvements as the MCS while the interaction effects did not reach statistical significance (p = 0.087), highlighting that the changes in mental component of HR-QoL are simultaneously influenced by fat mass and muscle mass. Nonetheless, this interaction effect needs to be interpreted with caution as it was not confirmed by our sensitivity analysis.

There are limitations to the current study. First, while the European Working Group for the Study of Sarcopenia proposed the presence of both low muscle mass and low muscle function (strength or performance) for the diagnosis of sarcopenia [48], our data did not include muscle functions. Because some studies have shown that dynapenia (i.e., loss of muscle function) but not low muscle mass is associated with functional limitations, disability, institutionalization and mortality [49], inclusion of muscular function is warranted in future studies. Additionally, there is a large heterogeneity in methods for determining fat mass and muscle mass, which is compounded by the heterogeneous body composition measures (e.g., bioelectrical impedance analysis, computed tomography, and DXA). We calculated the ASMI by adjusting muscle mass for BMI instead of height as the latter strongly correlates with BMI and identifies few individuals with low-muscle mass when BMI is high [50]. Indeed, in our cohort, the correlation between the muscle mass adjusted for height and BMI was significant (r = 0.503, p < 0.001). Our approach to determine ASMI has been indicated to detect sarcopenia in patients with higher BMI [51] and predict cardiometabolic risk factors in low muscle mass [52]. The cut-offs we used for the ASMI to separate high- and low- muscle mass were 0.580 and 0.877 for females and males, respectively. Our cut-offs were slightly higher than those previously reported by the FNIH: 0.512 for females and 0.789 for males [31]. The differences are probably due to younger age of our study participants. Second, although we divided participants into high fat and low fat based on FMI deciles, the mean BMI of females with low fat mass was 31.7 ± 2.6 kg/m^2^. For males, it was 31.0 ± 2.5 kg/m^2^ for low fat mass. It is important to note that the mean BMI of both sexes with low fat mass fell in the obesity category. Third, there were significant differences in baseline characteristic between participants who underwent DXA and those who did not and, thus, were excluded from our study. Participants who completed DXA were younger and metabolically healthier than those who did not undergo DXA. This limits the generalizability of our results to those who have more favorable cardiometabolic conditions. Lastly, because this is a retrospective analysis of existing data, it can only provide associative evidence, not causal.

## Conclusion

In conclusion, our sex-specific assessment of fat mass and muscle mass showed that low fat mass and low muscle mass are synergistically associated with higher A1C in females with type 2 diabetes, whereas high fat mass and low muscle mass independently contribute to other cardiovascular health risk factors regardless of sex, such as worse systolic BP, CRF, depression and HR-QoL scores. These findings suggest that strategies to increase muscle mass are necessary to reduce CVD risks in people living with type 2 diabetes, especially in females with low fat mass to control A1C. Our results highlight the complicated and sex-specific contribution of fat mass and muscle mass on CVD risk factors.

## Supplementary Information

**Additional file 1: Table S1**. Female, medication details. **Table S2**. Male, medication details.

## Data Availability

The data from Look AHEAD are available in the NIDDK Central Repository.

## Abbreviations

ANCOVA: Analysis of covariance
ASMI: Appendicular muscle mass index
A1C: Glycated hemoglobin A1C
BDI: Beck Depression Inventory
BMI: Body mass index
BP: Blood pressure
CRF: Cardiorespiratory fitness
CVD: Cardiovascular disease
DSE: Diabetes support and education
DXA: Dual-energy X-ray absorptiometry
FMI: Fat mass index
FNIH: Foundation for the National Institutes of Health
HDL-C: High-density lipoprotein cholesterol
HR-QoL: Health-related quality of life
ILI: Intensive lifestyle intervention
LDL-C: Low-density lipoprotein cholesterol
MCS: Mental component summary
NHANES: National Health and Nutrition Examination Survey
PCS: Physical component summary
SF-36: Medical Outcomes Study Short Form-36
STROBE: Strengthening the Reporting of Observational Studies in Epidemiology
