# Supplementary material for: Sex-specific associations of fat mass and muscle mass with cardiovascular disease risk factors in adults with type 2 diabetes living with overweight and obesity: secondary analysis of the Look AHEAD trial
Source: Cardiovasc Diabetol. 2022 Mar 15;21:40. doi: 10.1186/s12933-022-01468-x (PMC8925200; doi:10.1186/s12933-022-01468-x)
Supplement: Supplementary file 1 — Additional file 1: Table S1. Female, medication details. Table S2. Male, medication details. [file 12933_2022_1468_MOESM1_ESM.docx]

**Supplementary tables**

Female, medication details

|  | High-FMI | | Low-FMI | | High FMI  vs.  Low FMI,  p-value | High ASMI  vs.  Low ASMI,  p-value |
| --- | --- | --- | --- | --- | --- | --- |
|  | High  ASMI  (n=157) | Low  ASMI (n=272) | High  ASMI (n=272) | Low  ASMI (n=156) |  |  |
| Biguanide, n (%) | 95 (60.9) | 156 (57.4) | 168 (62.0) | 93 (59.6) | 0.460 | 0.308 |
| Sulfonylurea, n (%) | 67 (42.9) | 130 (47.8) | 108 (39.9) | 76 (48.7) | 0.388 | **0.036** |
| Insulin, n (%) | 27 (17.3) | 45 (16.5) | 41 (15.2) | 27 (17.3) | 0.734 | 0.734 |
| Rosiglitazone, n (%) | 14 (9.0) | 31 (11.4) | 20 (7.4) | 10 (6.4) | 0.071 | 0.403 |
| TZD, n (%) | 29 (18.6) | 58 (21.3) | 30 (11.1) | 24 (15.4) | **0.002** | **0.035** |
| Meglitinides, n (%) | 2 (1.3) | 6 (2.2) | 8 (3.0) | 2 (1.3) | 0.329 | 0.888 |
| Repaglinide, n (%) | 2(1.3) | 4 (1.5) | 2 (0.7) | 1 (0.6) | 0.225 | 0.948 |
| Statin, n (%) | 64 (40.8) | 89 (32.6) | 99 (36.5) | 58 (37.2) | 0.352 | 0.508 |
| ACE, n (%) | 53 (33.8) | 109 (39.9) | 107 (39.3) | 66 (42.3) | 0.711 | 0.368 |
| Alpha blocker, n (%) | 3 (1.9) | 8 (2.9) | 7 (2.6) | 1 (0.6) | 0.976 | 0.472 |
| Antidepressant, n (%) | 35 (22.3) | 55 (20.1) | 41 (15.1) | 16 (10.3) | **0.013** | 0.326 |
| Beta blocker, n (%) | 26 (16.6) | 46 (16.8) | 58 (21.3) | 25 (16.0) | 0.499 | 0.427 |
| Calcium channel blocker, n (%) | 31 (19.7) | 50 (18.3) | 42 (15.4) | 22 (14.1) | 0.273 | 0.841 |

Male, medication details

|  | High-FMI | | Low-FMI | | High FMI  vs.  Low FMI,  p-value | High ASMI  vs.  Low ASMI,  p-value |
| --- | --- | --- | --- | --- | --- | --- |
|  | High  ASMI  (n=72) | Low  ASMI (n=184) | High  ASMI (n=184) | Low  ASMI  (n=71) |  |  |
| Biguanide, n (%) | 47 (65.3) | 115 (62.5) | 119 (64.7) | 46 (64.8) | 0.828 | 0.558 |
| Sulfonylurea, n (%) | 45 (62.5) | 100 (54.3) | 93 (50.5) | 34 (47.9) | 0.759 | 0.350 |
| Insulin, n (%) | 9 (12.5) | 32 (17.4) | 29 (15.8) | 10 (14.1) | 0.054 | 0.613 |
| Rosiglitazone, n (%) | 10 (13.9) | 27 (14.7) | 25 (13.6) | 10 (14.1) | 0.210 | 0.599 |
| TZD, n (%) | 22 (30.6) | 51 (27.7) | 39 (21.2) | 14 (19.7) | **<0.001** | **0.013** |
| Meglitinides, n (%) | 3 (4.2) | 3 (1.6) | 10 95.4) | 1 (1.4) | 0.221 | 0.464 |
| Repaglinide, n (%) | 3 (4.2) | 1 (0.5) | 8 (4.3) | 0 (0.0) | 0.995 | 0.564 |
| Statin, n (%) | 41 (56.9) | 87 (47.3) | 96 (52.2) | 30 (42.3) | 0.565 | 0.452 |
| ACE, n (%) | 33 (45.8) | 79 (42.9) | 97 (52.7) | 30 (42.3) | 0.449 | 0.401 |
| Alpha blocker, n (%) | 10 (13.9) | 19 (10.3) | 20 (10.9) | 14 (19.7) | 0.880 | 0.674 |
| Antidepressant, n (%) | 11 (15.3) | 17 (9.2) | 17 (9.2) | 8 911.3) | 0.114 | 0.459 |
| Beta blocker, n (%) | 20 (27.8) | 43 (23.4) | 44 (23.9) | 13 (18.3) | 0.013 | 0.390 |
| Calcium channel blocker, n (%) | 12 (16.7) | 30 (16.3) | 45 (24.5) | 9 (12.7) | 0.983 | 0.804 |
